# Evidence‐to‐Decision Frameworks: Enhancing the Quality and Rigour of Guidelines and Recommendations

**DOI:** 10.1002/gin2.70078

**Published:** 2026-06-05

**Authors:** Jose F. Meneses‐Echavez, Pablo Alonso‐Coello, Gunn Vist, Javier Bracchiglione, Yang Song, Signe Flottorp, Sarah E. Rosenbaum

**Affiliations:** ^1^ Facultad de Cultura Física, Deporte y Recreación Universidad Santo Tomás Bogotá Colombia; ^2^ Department of Infectious Disease Control and Vaccines Norwegian Institute of Public Health Oslo Norway; ^3^ Institut de Recerca Sant Pau (IR SANT PAU) Sant Quintí Barcelona Spain; ^4^ Centro de Investigación Biomédica en Red de Epidemiología y Salud Pública (CIBERESP) Madrid Spain; ^5^ Centro Cochrane Iberoamericano Sant Antoni Maria Claret Barcelona Spain; ^6^ Cluster for Health and Social Care Interventions, Division of Health Services Norwegian Institute of Public Health Oslo Norway; ^7^ Interdisciplinary Centre for Health Studies (CIESAL) Universidad de Valparaíso Chile; ^8^ School of Medicine The Chinese Univeristy of Hong Kong‐Shenzhen Shenzhen China; ^9^ Centre for Epidemic Interventions Research (CEIR) Norwegian Institute of Public Health Oslo Norway

## Abstract

- The journey of guidelines being informed by evidence is less than 50 years old, but it has seen notable advances documented in various publication series and led by key organizations.
- Although Evidence‐to‐Decision (EtD) frameworks were officially born in 2016, since the early 2000s, groups like GRADE had begun to formulate key determinants of the recommendation‐formulation process.
- The EtD frameworks started with criteria such as the balance between desirable and undesirable outcomes, values and preferences, and resource use. Nowadays, they are extended to cover criteria like planetary health, human rights, and socio‐cultural acceptability.
- Experiences with the use of EtD frameworks are generally positive; they are flexible, facilitate multidisciplinary discussions, and bring rigor and transparency to the guidelines.
- More guidance is required on how to use EtD frameworks, as well as more research to understand how they can lead to better guidelines, and more recently, how artificial intelligence can responsibly help navigate this path.

1

This commentary briefly describes the evolvement of guideline development, leading to the introduction of Evidence‐to‐Decision (EtD) frameworks. It then outlines key existing frameworks and summarises evidence on users' experiences. Finally, it discusses how EtD frameworks may contribute to improved guideline quality and reflects on future directions, including artificial intelligence.

## 2 Evolution of Guideline Development Methods: A Historical Perspective

Health guidelines consist of recommendations for improving health and well‐being [1, 2]. In the early 20th century, guidelines were largely based on expert opinion, with limited transparent consideration of the quality of the evidence [3]. While the World Health Organisation (WHO) began issuing formal guidelines in 1977 [4], the first guideline recognised as evidence‐based was published in 1979 by the Canadian Task Force [5]. These initiatives influenced the establishment of similar organisations worldwide, such as the U.S. Preventive Services Task Force in 1984 [6], and contributed to the development of evidence‐informed guidelines by the WHO [7], the National Institute for Health and Care Excellence (NICE) [8], and other health agencies [7].

Other advances in the late 20th century included the emergence of Evidence‐Based Medicine (EBM) in the 1990s [9] and the establishment of Cochrane in 1993. In 2000, the GRADE (Grading of Recommendations Assessment, Development, and Evaluation) Working Group was founded. The first article describing GRADE's approach to grading the quality of evidence and strength of recommendations was published in 2004 [10], followed by a series of methodological papers in the *British Medical Journal* in 2008 targeting guideline developers and users [11]. By 2007, WHO had already begun incorporating the GRADE approach into some of its guidelines [7, 12]. By the 2010s, a rapid proliferation of guidelines was accompanied by increased concerns about conflicts of interest and demands for credibility. This accelerated the adoption of standards such as Clinical Practice Guidelines We Can Trust [13] and the WHO Handbook for Guideline Development [14].

This context sparked the use of frameworks to ensure all relevant criteria were systematically considered when moving from evidence to decisions, a process that ultimately led to the emergence of Evidence‐to‐Decision (EtD) frameworks.

### 2.1 From Evidence to Action: The Foundations of EtD Frameworks

**Figure 1:**
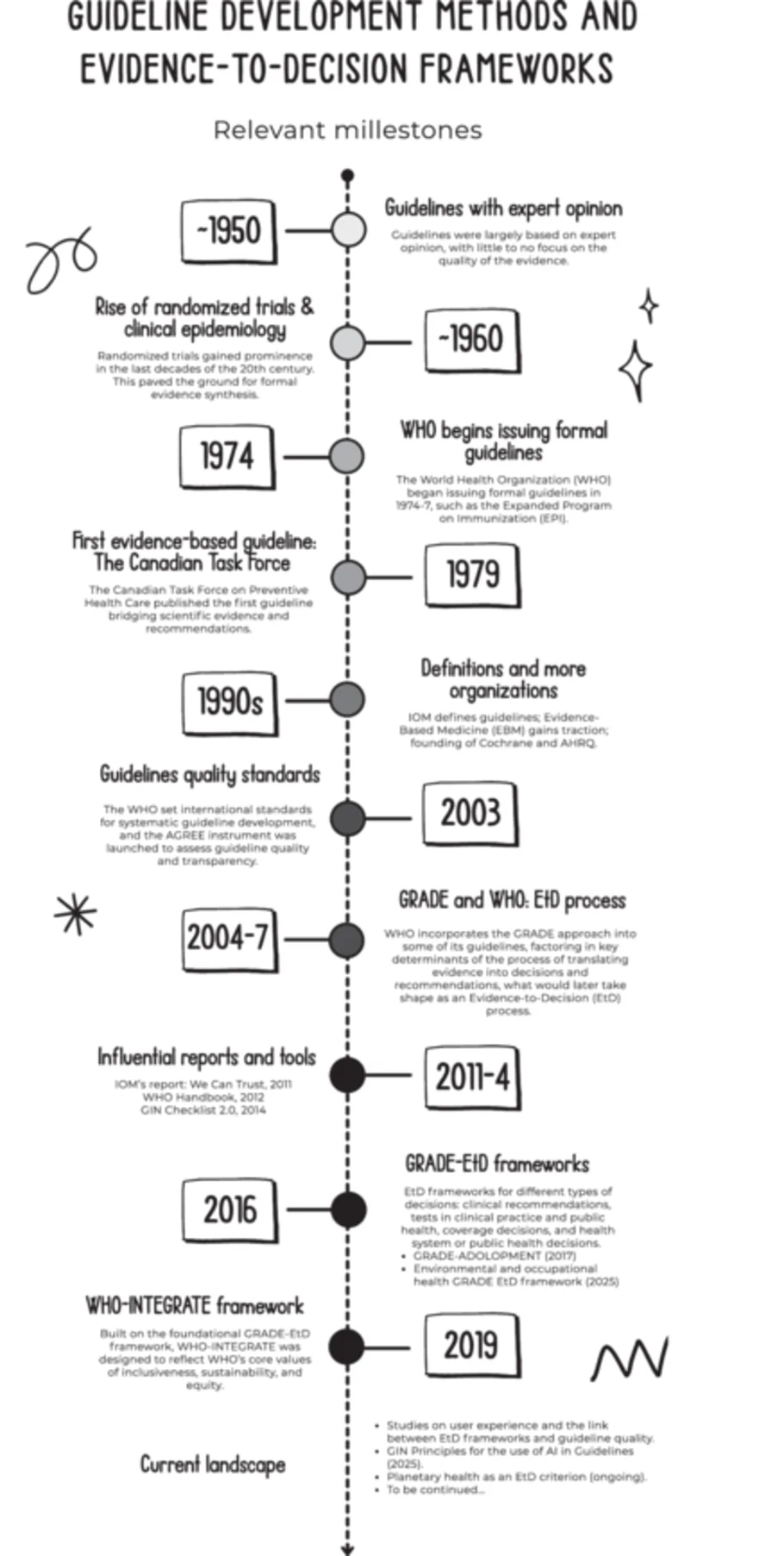
Timeline of guideline development methods and evidence‐to‐decision frameworks.

In 2011, the GRADE Working Group began publishing a series of articles in the *Journal of Clinical Epidemiology*, addressing various aspects of guideline development [15]. In 2013, the group outlined a set of key determinants of the direction and strength of recommendations, including the balance between desirable and undesirable outcomes (estimated effects), with consideration of values and preferences, confidence in those estimates, uncertainty or variability in values and preferences, and resource use [16]. The GRADE Working Group emphasised that guideline panels should integrate these determinants when formulating recommendations to ensure transparency and consistency. This work led to the introduction of what the authors termed an “*Evidence to recommendation framework*” [16, 17] (Figure 1).

The term “Evidence‐to‐Decision (EtD) framework” is often used as an umbrella concept due to a lack of a universally accepted definition. We define it as a structured approach designed to support recommendations or decisions by explicitly outlining key criteria, integrating diverse perspectives, and systematically considering relevant evidence. In practice, this approach involves the use of standardised methodologies to identify the key criteria informing a recommendation, systematically locate and synthesise relevant evidence, and present that evidence to interest‐holders in a transparent deliberative process.

Overall, EtD frameworks support panel judgments for each guideline criterion and serve as a *roadmap* for deliberations on the evidence underpinning guideline recommendations [18]. The frameworks facilitate interest‐holder engagement and the systematic considerations of real‐world factors, such as resource use, feasibility, and equity. Equally important is their role in promoting communication with end‐users, thereby supporting the adaptation and implementation of recommendations across diverse contexts [19, 20, 21, 22].

## 3 Types of EtD Frameworks and Their Applications

EtD frameworks have become a central tool for bringing transparency and accountability to guidelines in the past two decades. Although they share common principles, they differ in their purpose, the criteria they include, and their areas of application.

### 3.1 GRADE‐Evidence‐to‐Decision (GRADE‐EtD) Frameworks

The most influential development occurred within the DECIDE project, which was led by the GRADE Working Group and funded by the European Union [23]. The project's main outcome was the development of the GRADE‐EtD frameworks [24], created through an iterative process involving extensive input from experts and users [22]. These frameworks are constructed around a set of proposed key criteria: problem prioritisation, balance of desirable and undesirable effects, certainty of the evidence, values and preferences, resource use, equity, acceptability, and feasibility [19, 25, 26, 27]. The group developed adapted versions for different types of recommendations and decisions, including for clinical practice [19], coverage [26], diagnostic tests [28], public health and health system [25], multiple comparisons [29], and guideline adaptation [30]. The GRADE Working Group is leading the GRADE Planetary Health Project, which provides guidance on integrating planetary health into health guidelines [31].

### 3.2 Other EtD Frameworks

Numerous organisations have developed variations of EtD frameworks over the past decades [32, 33]. EtD frameworks now exist for clinical practice [19], immunisation practices [34, 35], environmental and occupational health [27], healthcare coverage decisions [26], health systems and public health [25], and economic evaluations [36]. Likewise, organisations such as NICE have developed frameworks that apply to a broader range of decisions [37, 38]. Despite their differences, most of these frameworks share similar development processes, including literature reviews, iterative consensus with end‐users and interest‐holders, and piloting [22, 39].

### 3.3 WHO‐INTEGRATE Framework

In 2019, the WHO introduced the WHO‐INTEGRATE EtD framework to enhance decision‐making for complex health interventions. WHO‐INTEGRATE sought to make societal, environmental, ethical, and cultural considerations more explicit within the decision‐making process [33, 40]. Developed in alignment with the broader GRADE‐EtD framework, WHO‐INTEGRATE reflects WHO's emphasis on inclusiveness, sustainability, and equity [40].

### 3.4 Comparison of Criteria Across EtD Frameworks

The EtD frameworks differ in the number and nature of the criteria they consider [30]. Some frameworks, such as the GRADE‐EtD frameworks [24] and WHO‐INTEGRATE framework [40] cover a broad set that includes balance of benefits and harms, certainty of the evidence, values and preferences, resource use, acceptability and feasibility. Others incorporate additional criteria such as sustainability, transferability, or social impact [40]. In any case, the choice of a framework, as well as the weight given to each criterion, depends on the field, the purpose of the guideline, and the perspective of the development panel. Table 1 summarises the main criteria considered by the GRADE, WHO‐INTEGRATE, and other frameworks. A more comprehensive overview of EtD frameworks can be found in previous work [32, 48].

**Table 1:** List of EtD criteria considered by GRADE, WHO‐INTEGRATE and other EtD frameworks.

EtD framework	Criteria
GRADE‐EtD framework [19]	Problem priority
	Desirable effects
	Undesirable effects
	Certainty of evidence of effects
	Values
	Balance of effects
	Resources required
	Certainty of evidence of required resources
	Cost effectiveness
	Equity
	Acceptability
	Feasibility
WHO‐INTEGRATE framework [40]	Balance of health benefits and harms
	Human rights and sociocultural acceptability
	Health equity, equality, and non‐discrimination
	Societal implications
	Financial and economic considerations
	Feasibility and health system considerations
	Quality of evidence*
Other frameworks**	Sustainability*** [40, 41, 42, 43]
	Evidence gaps [44]
	Interest‐holder engagement [41, 45, 46, 47]
	Transferability [43]
	Complementarities and interactions among strategies [42]
	Implications for the course of the pandemic and its impact on health [38]

* Considered as a meta‐criterion, applicable to all criteria.

** Criteria not explicitly considered by GRADE and WHO‐INTEGRATE, but present in other frameworks.

*** Sustainability encompasses ecological, economic, and social considerations.

The EtD frameworks have broadened the evidence considered in guidelines. Whereas criteria such as desirable and undesirable effects often rely on quantitative studies, others, such as values and preferences and acceptability, rely more heavily on qualitative evidence. These differences underscore the importance of transparent reporting of EtD processes to ensure clarity, accountability, and reproducibility in guidelines.

GRADE‐EtD frameworks are designed to be flexible and should be adapted to fit both the guideline development process and the specific context in which recommendations will be implemented [19, 21]. Organisations may choose to merge certain criteria or leave others unaddressed. Such prioritisation of EtD criteria may depend on available resources or normative values within the guideline context. Regardless of the approach, and for the sake of transparency, guideline developers must clearly report which EtD criteria were prioritised and the reasons for doing so [19, 21].

## 4 Users' Views on the Use of EtD Frameworks

The successful use of EtD frameworks depends not only on their design but also on how users experience them in practice [49]. During deliberations, an experienced panel chair is vital to maintain balance, preventing domineering voices from overshadowing discussions, and ensuring focused and productive deliberations [21, 33, 50]. Guideline development groups often require training to complete frameworks, use electronic tools, and apply methods for grading the evidence, including situations where evidence is scarce, missing or of low certainty.

EtD frameworks present challenges. Some users perceive them as time‐consuming or overly comprehensive [21]. Others struggle to distinguish closely related criteria (e.g., acceptability vs. feasibility [21]). People may find criteria and sub‐criteria confusing, such as equity, equality, and non‐discrimination [33, 50]. Limited training opportunities further hinder full implementation and the quality of reporting [33, 50].

Evidence from user studies, however, shows important benefits. Our team has recently identified 11 studies exploring users' experiences with the EtD frameworks [51], all of which covered only two of the 15 EtD frameworks we identified in the public health literature. Eight of these studies examined experiences with the GRADE‐EtD framework [18, 21, 52, 53, 54, 55, 56, 57], while three explored the WHO‐INTEGRATE framework [50, 58, 59].

Users valued the structured and transparent approach, access to evidence summaries for each criterion, and the opportunity for meaningful participation [21, 50]. These features facilitated multidisciplinary discussions, broadened the range of contextual factors considered beyond clinical effects, and fostered accountability [21, 50]. Overall, users' experiences suggest that EtD frameworks, when applied flexibly and transparently, can support rather than obstruct deliberation and enhance the rigour and legitimacy of guidelines [21, 50].

## 5 Do Evidence‐to‐Decision Frameworks Lead to Better Guidelines?

A recent study examined the association between the use of EtD frameworks and the quality of guidelines [60], evaluating 66 guidelines and their recommendations, published across 17 countries. Guidelines that employed an EtD framework scored higher across all domains of both AGREE‐II and AGREE‐REX compared with those that did not. The EtD frameworks supported more systematic appraisal of evidence, facilitated interest‐holder engagement, and promoted explicit links between evidence and recommendations, thereby improving clarity, transparency, and applicability. More representative research is warranted before confirming this, particularly guidelines from Asia, Latin America, and Africa.

## 6 Final Reflections and the Road Ahead

For EtD frameworks to continue supporting the advancement of more transparent and evidence‐informed decision‐making, significant efforts are required. The available guidance must be improved, not only in terms of the suggested criteria and their definitions but also in ensuring greater flexibility within these frameworks [21, 61]. Framework developers should provide practical examples of their application and scenarios in which they may be adapted (e.g., combining certain criteria or incorporating alternative types of evidence). Recent research has explored the potential of simulation to support people both in developing frameworks and using them in group deliberations [62, 63].

The use of EtD frameworks must align with the needs of the local context where recommendations will be implemented [19, 22, 40, 56]. Failure to consider this can lead to guidelines that are disconnected from real‐world settings. Additionally, EtD frameworks may unintentionally lead guideline development groups to prioritise quantitative over qualitative evidence [64, 65, 66].

Further research is needed on the impact of using EtD frameworks [60]. Studies exploring the impact of the EtD frameworks and user experiences might improve their generalisability by including a broader range of guidelines and participants (including people without expertise in GRADE methods), as well as real‐world observation studies. Research is needed on the use of EtD frameworks in non‐clinical fields, such as social welfare and environmental health [27, 67].

Artificial Intelligence (AI) may help streamline the use of EtD frameworks by accelerating the retrieval of evidence needed to evaluate framework criteria [68, 69, 70]. However, AI use in evidence synthesis is still exploratory, with scarce evidence from validation studies that have mixed findings and heterogeneous methodologies [68, 71, 72]. To date, the evidence does not support the use of AI in evidence synthesis without human involvement or oversight [73, 74].

The Guidelines International Network (GIN) recently launched principles for the development and use of AI tools in the guideline enterprise [75]. These encompass transparent reporting; an assessment of the limitations, risks, and benefits of AI tools; documented validation of the performance of AI tools; and accountability, ethics, and compliance with human rights and legal frameworks [75]. Cochrane has recently published recommendations for responsible and transparent use of AI in evidence synthesis [76]. Every guideline development group is ultimately responsible for the use and effects of AI in guideline development, ensuring adherence to legal and ethical standards [74, 75].

Any use of AI should take into account the serious concerns regarding the lack of transparency, potential human rights violations associated with large language models [77, 78], and their environmental impact [79]. A recent evaluation of nine different language models demonstrated that these generate divergent and biased recommendations, even when provided with identical clinical data, for patients labelled as Black or LGBTQIA+ (lesbian, gay, bisexual, transgender, queer or questioning, intersex, asexual, and more) [80]. Moreover, language models tend to prescribe more advanced imaging tests for cases labelled as high‐income, while those labelled as low‐ or middle‐income are frequently restricted to basic or no additional testing [80]. Fake citations generated by AI have already been published by national public health authorities [81].

A non‐responsible uptake of AI might lead guidelines to reproduce well‐documented biases, as well as unidentified biases and prejudices embedded in the data on which large language models are trained. These harms would ruin any EtD framework and make guidelines prone to biased recommendations, thereby undermining their overarching purpose of contributing to a better world.

## Author Contributions

**Jose F Meneses‐Echavez:** conceptualisation, investigation, writing – original draft, methodology, writing – review and editing, project administration, supervision. **Pablo Alonso‐Coello:** conceptualisation, investigation, writing – original draft, writing – review and editing. **Gunn Vist:** conceptualisation, writing – original draft, writing – review and editing. **Javier Bracchiglione:** conceptualisation, writing – original draft, writing – review and editing, methodology. **Yang Song:** conceptualisation, writing – original draft, writing – review and editing, methodology. **Signe Flottorp:** conceptualisation, writing – original draft, writing – review and editing, methodology. **Sarah E. Rosenbaum:** conceptualisation, writing – original draft, writing – review and editing, methodology.

## Funding

The authors have nothing to report.

## Conflicts of Interest

Some authors of this commentary have been involved in the development of the GRADE Evidence‐to‐Decision (EtD) frameworks; all authors are members of the GRADE Working Group.

## Data Availability

The data that support the findings of this study are available from the corresponding author upon reasonable request.
